# Epidemic Influenza

**Published:** 1891-11

**Authors:** 


EPIDEMIC INFLUENZA; Notes on its origin and methods of spread. By Richard Sisley, M.D. Longmans, Green & Co., London and New York, 1891. $2.50.

Influenza is treated from the standpoint of human medicine, and the author gives the various nomenclature; he then defines the disease; gives the origin, the method of its spread, and historical sketch of the epidemic in various countries and under diverse conditions, which lead to the conclusion of the contagiousness of its nature. Chapter XIII. treats of influenza in animals, but offers no proof that there is any identity or even analogy between the disease of man and that of the horse, known by the same name, beyond a few opinions without facts. He seems to confuse strangles with influenza. It is an interesting book and well worth reading.

